# Recurrent Heatwaves Slow Down the Recovery of a Phytoplankton Community

**DOI:** 10.1002/ece3.70539

**Published:** 2024-12-11

**Authors:** Francesco Polazzo, Markus Hermann, Melina Crettaz‐Minaglia, Andreu Rico

**Affiliations:** ^1^ Department of Evolutionary Biology and Environmental Studies University of Zurich Zurich Switzerland; ^2^ Department of Aquatic Ecology Eawag, Swiss Federal Institute of Aquatic Science and Technology Dübendorf Switzerland; ^3^ IMDEA Water Institute Science and Technology Campus of the University of Alcalá Alcalá de Henares Madrid Spain; ^4^ Cavanilles Institute of Biodiversity and Evolutionary Biology University of Valencia Paterna Valencia Spain

**Keywords:** community rescue, critical slow down, ecological stability, heatwaves, resilience

## Abstract

Heatwaves (HWs) are predicted to increase in frequency and severity due to climate change. Yet, there is limited information about how ecological resilience of aquatic communities is going to be impacted by recurrent HWs. Here, we used data from an outdoor freshwater mesocosm experiment where a semi‐natural phytoplankton community was exposed to three subsequent HWs. The data were used to test two different hypotheses regarding community and ecosystem responses to recurrent perturbations: critical slowing down and rescue. Slowing down would determine a reduction in resilience and eventually a community or ecosystem collapse, whereas rescue would increase community or ecosystem resilience and maintain stable community and ecosystem properties. The results of our experiment showed evidence for critical slowing down, but not for community or ecosystem rescue. The recovery capacity of phytoplankton biomass and dissolved oxygen gradually decreased after the first two HWs and sharply declined after the third one. The decline in these community and ecosystem properties were linked to a significant compositional turnover in the phytoplankton community. Although we did not find evidence for a transition into an alternative stable state, our results provide insights into how the overall resilience of a phytoplankton community may decline in the presence of recurrent heatwaves. Thus, we highlight the importance of monitoring the slowing down of recovery of aquatic communities experiencing repeated exposure to severe perturbations.

## Graphical abstract

Our study evaluates how repeated heatwaves affect the ecological resilience of freshwater communities. It shows that repeated heatwaves result in a critical slowing down of phytoplankton communities and underscore potential for reduced overall resilience due to repeated heatwave events.

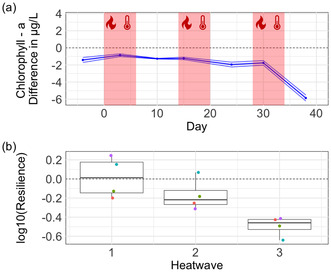

## 1 Introduction

Climate change is an urgent global challenge characterised by significant alterations in weather patterns (IPCC 2023). One of the most concerning aspects of climate change is the increasing frequency and intensity of extreme events, such as hurricanes, floods, droughts, and and heatwaves (Fischer, Sippel, and Knutti 2021). Particularly, heatwaves (HWs) have been projected to increase in frequency and severity globally (Perkins, Alexander, and Nairn 2012), affecting the freshwater realm (Woolway et al. 2021, 2022). Several studies show that HWs can detrimentally impact the diversity and functioning of freshwater ecosystems (Correa‐Araneda et al. 2020; Mouthon and Daufresne 2006; Polazzo et al. 2022; Woodward et al. 2016). Yet, the effects of HWs on ecological stability have hardly been assessed (Polazzo et al. 2022). The few studies that investigated the effects of HWs on ecological stability have shown that HWs can negatively affect several dimensions of functional and compositional stability of freshwater populations and communities, including resistance, recovery and temporal stability (Polazzo et al. 2023; Ross et al. 2022).

Additionally, HWs have been reported to decrease the resilience of aquatic ecosystems, causing critical transitions to alternative stable states (Bertani, Primicerio, and Rossetti 2016; Meunier, Hacker, and Menge 2024; Turner et al. 2020; Wernberg et al. 2016). In this context, resilience is defined as the ability of a system to absorb perturbations without transitioning to an alternative equilibrium or stable state (Holling 1973). However, evidence for HW‐driven abrupt shifts or collapses is limited in freshwater systems (Bertani, Primicerio, and Rossetti 2016; Filiz et al. 2020; Polazzo et al. 2022). Scarce support for HW related collapse may be linked to the fact that most empirical studies analyse the impact of a single HW event (Polazzo et al. 2022), and the few available studies considering recurrent HWs have not focused on assessing cumulative effects on ecological resilience (Hermann, Peeters, and Van den Brink 2023; Hermann et al. 2024). Yet, with heatwaves expected to become more common in the future, concerns have been raised about whether and how natural communities can sustain multiple recurring HWs.

How repeated perturbations affect the resilience of a system can be understood through two major ecological frameworks: critical slowing down and community or ecosystem rescue. Critical slowing down is the process by which functional and/or structural recovery of communities decreases when they are close to a tipping point because the internal stabilising forces of the community become weaker (Veraart et al. 2012). The exact shifting point is notoriously difficult to predict, as ecosystems exhibit complex, nonlinear interactions among various biotic and abiotic components, where small changes can lead to disproportionate effects (van Nes and Scheffer 2007). Therefore, the focus has shifted to deducing processes from patterns. This involves identifying observable signals in measurable endpoints of a biological system that indicate changes in the system's behaviour, which may result in a critical transition. In the last two decades, the phenomenon known as critical slowing down has been indicated as a possible early warning signal (EWS) of an approaching abrupt shift, derived from dynamic systems theory (Rietkerk et al. 1996; Strogatz 2019). EWSs are based on the idea that recovery rates from repeated perturbations tend to zero as a system approaches a transition point (Rietkerk et al. 1996; Strogatz 2019; Veraart et al. 2012).

On the other hand, community and ecosystem rescue theory suggests that ecological or evolutionary processes may restore recovery under recurrent stressful conditions, thereby preventing community or ecosystem collapse (Bell and Gonzalez 2011; Carlson, Cunningham, and Westley 2014; Samani and Bell 2010). Although empirical examples of community rescue are scarce (Fugère et al. 2020), it is considered a key mechanism that enhances community resistance and helps maintaining aggregate community properties, such as biomass, under stressful conditions. Both frameworks are plausible and have been documented in communities undergoing repeated perturbations (Fugère et al. 2020; Veraart et al. 2012). However, to the best of our knowledge, they have not been applied to assess the impacts of recurrent heatwaves in freshwater ecosystems. The extent to which critical slowing down or rescue occurs in aquatic ecosystems exposed to recurrent HWs is therefore unknown.

The aim of this study was to assess the role of critical slowing down or rescue in explaining the response of phytoplankton biomass and dissolved oxygen when experiencing recurrent HWs. For this, we used data coming from an outdoor pond mesocosm experiment where a semi‐natural phytoplankton community was exposed to three subsequent heatwaves separated by 1 week at ambient temperature. Such experimental design allowed us to assess the impacts of each of the three HWs on phytoplankton biomass, composition and dissolved oxygen concentration, as well as the change in the short‐term recovery of these properties. We hypothesised that if rescue prevails, the first HW will determine a decline in community and ecosystem properties, which will be followed by a compositional change of the phytoplankton community that promotes stress‐tolerant species and/or genetic adaptation (Fugère et al. 2020). This new community might be more resistant to a following HW, and thus determine higher community stability to future HWs. Conversely, if critical slowing down prevails, the stress accumulation due to recurrent HWs will gradually reduce the recovery rate of the phytoplankton community after each HW, reducing community resilience, and driving the community to a collapse.

## 2 Materials and Methods

### 2.1 Experimental Design

An outdoor mesocosm experiment was performed at the facilities of the IMDEA Water Institute (Alcalá de Henares, Madrid, Spain) between April and July of 2021. The original experiment comprised 24 mesocosms, different temperature regimes and an additional chemical stressor (Hermann et al. 2024). Here, we used a subset of the original data, only looking at the recurring HW treatment. For this, 8 mesocosms were used. All mesocosm were round, glass fibre ponds with a diameter of 1.2 m, a total depth of 1.2 m and a volume of approximately 1000 L. Each mesocosm contained a 30–40 cm layer of silty‐sand sediment sourced from the area around the institute and was filled with 850 L of freshwater from an artificial pond at the research facility. To account for water loss due to evaporation, we placed tap water in plastic buckets biweekly and exposed it to outdoor conditions for several days to reduce chlorine levels before refilling the mesocosms to their original volume of 850 L. The biological community of the mesocosms was largely derived from the artificial pond and was composed of phytoplankton, zooplankton, and macroinvertebrates. Additional macroinvertebrates were collected independently of size and sex from the Henares River near Humanes, Guadalajara (Spain), and evenly distributed in the mesocosms. The biological community was allowed to establish and adapt to the experimental units for 3 months prior to the start of the experiment. During this period, water was randomly exchanged between mesocosms to homogenise the biological communities. Detailed information on the experimental units and the stocking of the biological communities can be found in Hermann et al. (2024).

**FIGURE 1:**
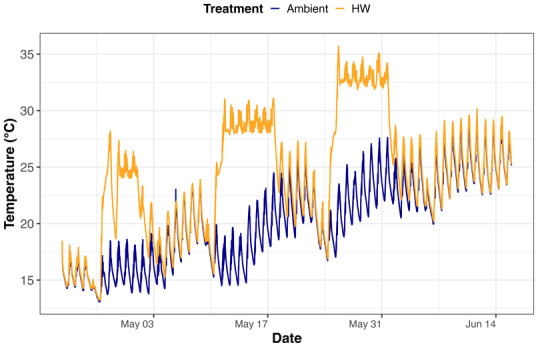
Water temperature dynamics over time in ambient mesocosms (blue line) and HW mesocosms (orange line). The drop in temperature in the HW treatment during the first HW was caused by a technical failure of the TENTACLE machinery during few hours.

Four mesocosms were used to simulate the HW scenario (*n* = 4), which was formed by three repeated HWs (Figure 1), while the remaining four mesocosms were kept at ambient temperature for the whole experimental duration and were used as temperature controls (*n* = 4). The HW treatment consisted of three HWs lasting 7 days each and separated one another by 7 days of ambient temperature. In the HW treatment, the temperature was +8°C above the control temperature, meaning that the absolute temperature of the HWs increased progressively from the first to the third HW as the mean water temperature in the control mesocosms increased because of seasonality (i.e., from spring to summer). The duration of the HW treatment was chosen as it is comparable to the average duration of recorded lake heatwaves, which typically last for 7.7 ± 0.4 days, while the intensity (+8°C) was selected based on projections of future HW intensity for the end of the century (IPCC 2021; Woolway et al. 2021).

All temperature manipulations and recordings were carried out using a transportable temperature and heatwave control device (TENTACLE) applicable for aquatic micro‐and mesocosm experiments (Hermann et al. 2022). Additionally, we placed a Hobo logger (Onset Computer Corporation, Bourne, MA, USA) in mesocosms undergoing both temperature treatments, the ambient temperature and recurrent HWs, to have an independent water temperature measurement.

### 2.2 Phytoplankton Sampling, Biomass Quantification, and Photosynthetic Activity

The phytoplankton community was sampled on days −4, 3, 10, 15, 24, 30, and 38, relative to the start of the first HW. Depth‐integrated water samples were collected from each mesocosm until a total volume of 5 L was obtained. Next, the sample was homogenised, and 250 mL of this water sample were introduced into amber glass bottles. The samples were preserved with 10% of Lugol's iodine solution. Phytoplankton taxa were identified and counted following the methods described by Rice et al. (2012), with small modifications. The 250 mL sample was allowed to sink (following an approach like the Utermöhl method). Then 1 mL sub‐samples of concentrated sample were taken and counted to a total of 400 cells or colonies, which corresponded to about 5–15 mL of subsample depending on the algae density. After the phytoplankton taxa were identified, they were counted by means of an inverted microscope and a Sedgewick‐Rafter counting cell (Graticules Optics). Finally, the counted cell or colony abundances were re‐calculated to number of cells per L of mesocosm water.

Every phytoplankton taxon was digitally photographed with scale reference using a Samsung 12 mp camera (4032 × 3024, JPG format), and measured using the Image J software (Schneider, Rasband, and Eliceiri 2012). The biovolume (μm^3^/org) of the phytoplankton cells was calculated using geometric models according to Hillebrand et al. (1999) and Sun and Liu (2003). Biovolume was transformed to fresh weight using the following ratio 106 μm^3^ = 1 μg, assuming that the specific density of the counted cells was the same as that of water, 1 kg/L (CEN 2006).

We also quantified chlorophyll *a* (chl *a*; μg/L) as a proxy for phytoplankton photosynthetic activity. Chl *a* was measured in situ on days −4, 3, 7, 10, 15, 24, 30 and 38 by using a portable multi‐meter (YSI Pro DSS 626,973–01). Calibration was established by using a regression model between rhodamine standard and chlorophyll‐a concentrations with temperature corrections.

### 2.3 Ecosystem Property

Dissolved oxygen (DO; mg/L) was measured as ecosystem property during the experimental period. Oxygen is essential to all aerobic organisms, and its dynamics in freshwater involves interconnected physical and biological processes that form the basis of the functioning of freshwater ecosystems. DO was measured on days −4, 3, 7, 10, 15, 24, 30, and 38. DO was measured in situ by using a portable multi‐meter (YSI Pro DSS 626,973–01), which was previously calibrated based on the Winkler method.

### 2.4 Zooplankton Sampling and Biomass Quantification

Since phytoplankton biomass dynamics can be heavily influenced by zooplankton grazing activity (Huỳnh et al. 2024), we also analysed zooplankton biomass and compositional dynamics. Zooplankton were sampled from each mesocosm on days −4, 10, 24 and 38 relative to the start of the first HW using a PVC tube. Depth‐integrated samples were collected from the mesocosm until a total volume of 5 L was obtained. The entire sample was then concentrated into a 100 mL polyethylene bottle using a 55 μm zooplankton net, preserved with Lugol's solution, and stored in the dark in the laboratory for species identification. A binocular microscope (Olympus SZX7) was used to examine and count all individuals from the Cladocera, Copepoda, and Ostracoda taxa (macro‐zooplankton). To analyse micro‐zooplankton, 1 mL subsamples were taken from the concentrated samples, and counts were adjusted to individuals per litre. Micro‐zooplankton (primarily Rotifera and naupliar stages of Copepoda) were identified and counted using a microscope (Olympus CX41).

Every zooplankton taxon was digitally photographed and measured as described above for the phytoplankton taxa. The biovolume (μm^3^/individual) of the zooplankton individuals was calculated using geometric models according to Alcaraz et al. (2003). Biovolume was transformed to fresh weight using the following ratio 1 μg = 106 μm^3^, assuming that the specific density of water was 1 kg/L (CEN 2006).

### 2.5 Statistical Analyses

To investigate the effect of the HWs on DO, chl *a*, phytoplankton and zooplankton biomass in the mesocosms, we employed a linear mixed‐effects model (LMM) using the *lmer* function from the “*lme4*” package (Bates et al. 2015). The model included HW (a factor with two levels: HW or Control), time and their interaction as fixed effects, with mesocosm identifier as a random effect to account for the repeated measures within each mesocosm. Since water temperature directly affect oxygen solubility in water, we included in the model only values measured in days when HWs were not occurring (i.e., when all mesocosms were at ambient temperature). The model diagnostics were performed using the “check_model” function from the “performance” package (Lüdecke, Makowski, and Waggoner 2020) to visually inspect that the assumptions of the LMM were met. We log‐transformed the raw data of DO and chlorophyll‐a concentration to meet the model's assumptions. When an interaction effect between HW and time was found we performed a post hoc comparison across different days using the “emmeans” package (Lenth et al. 2024) to perform an estimated marginal means (EMMs) analysis.

To quantify whether there was a change in the resilience of the evaluated variable after each HW, we quantified resilience following the method proposed by Baert et al. (2016). Resilience was calculated as the proportional change in deviation in the variable between the HW treatment and the control between the sampling before each HW (days −4, 10, 24) and the sampling after each HW (days 10, 24, 38).

(1) $$\text{Resilience}=\frac{\left|X_{\text{control}\_\mathrm{pre}\_\mathrm{HW}}-X_{\text{control}\_\text{post}\_\mathrm{HW}}\right|}{\left|X_{\mathrm{HW}\_\mathrm{pre}\_\mathrm{HW}}-X_{\mathrm{HW}\_\text{post}\_\mathrm{HW}}\right|}$$

where $X_{\text{control}\_\mathrm{pre}\_\mathrm{HW}}$ represents the value of the variable in the control on the sampling before the beginning of the HW, and $X_{\text{control}\_\text{post}\_\mathrm{HW}}$ represents the value of the variable in the control on the sampling day after the beginning of the HW. $X_{\mathrm{HW}\_\mathrm{pre}\_\mathrm{HW}}$ represents the value of the variable in the mesocosms experiencing the HW treatment the sampling before the beginning of the HW, and $X_{\mathrm{HW}\_\mathrm{pre}\_\mathrm{HW}}$ represents the value of the variable in the mesocosms experiencing the HW treatment on the sampling after the end of the HW. This resulted in 3 values of resilience for DO, chl *a*, and phytoplankton biomass, calculated respectively for the time periods: day −4–10, 10–24, and 24–38, and corresponding to the three consecutive HWs.

Resilience is > 1 when differences between the before and after situation regarding the HW are larger in the control than in the HW treatment, and < 1 otherwise. Please note that in the figures below resilience was log10 transformed, so the benchmark for the resilience classification becomes 0 instead of 1. Thus, in case of critical slowing down, we expect negative resilience values as there is an erosion of resilience with each consecutive HW. In case of rescue, we expect resilience to progressively move from negative to positive after each HW, which would suggest an increase in resilience due to previously experienced stress.

Both critical slowing down and rescue are mechanistically underpinned by compositional changes in the evaluated community. To quantify changes in phytoplankton community composition, we performed a non‐parametric permutational multivariate analysis of variance (PERMANOVA), using the function “adonis2” of the R package “vegan” (Oksanen et al. 2019) with 999 permutations and based on Bray‐Curtis distances calculated on the biomass of phytoplankton taxa. We repeated the same analysis for zooplankton.

To further assess the dissimilarity in community composition between the control and the HW treatment, the Similarity Percentage (SIMPER) analysis was conducted using the function “simper” of the package “vegan” (Oksanen et al. 2019). This method identifies the contributions of individual taxa to the dissimilarity between groups to the overall Bray–Curtis dissimilarity.

All statistical analysis and figures were done in R (R version 4.2.2; R Core Team 2022). Data and code to reproduce the analysis and figures are available at https://github.com/FrancescoPola/rescue_critical_slowing, and permanently deposited in Zenodo.

## 3 Results

### 3.1 Temperature Manipulations

Throughout the whole experiment, the average ambient water temperature ranged between 13°C and 30°C, with a mean temperature of 20°C (Figure 1). Except for a few temporary declining temperature periods, the ambient water temperature gradually increased during the experiment. The average HW water temperature was 24°C and ranged between 13°C and 36°C.

### 3.2 Dissolved Oxygen

**FIGURE 2:**
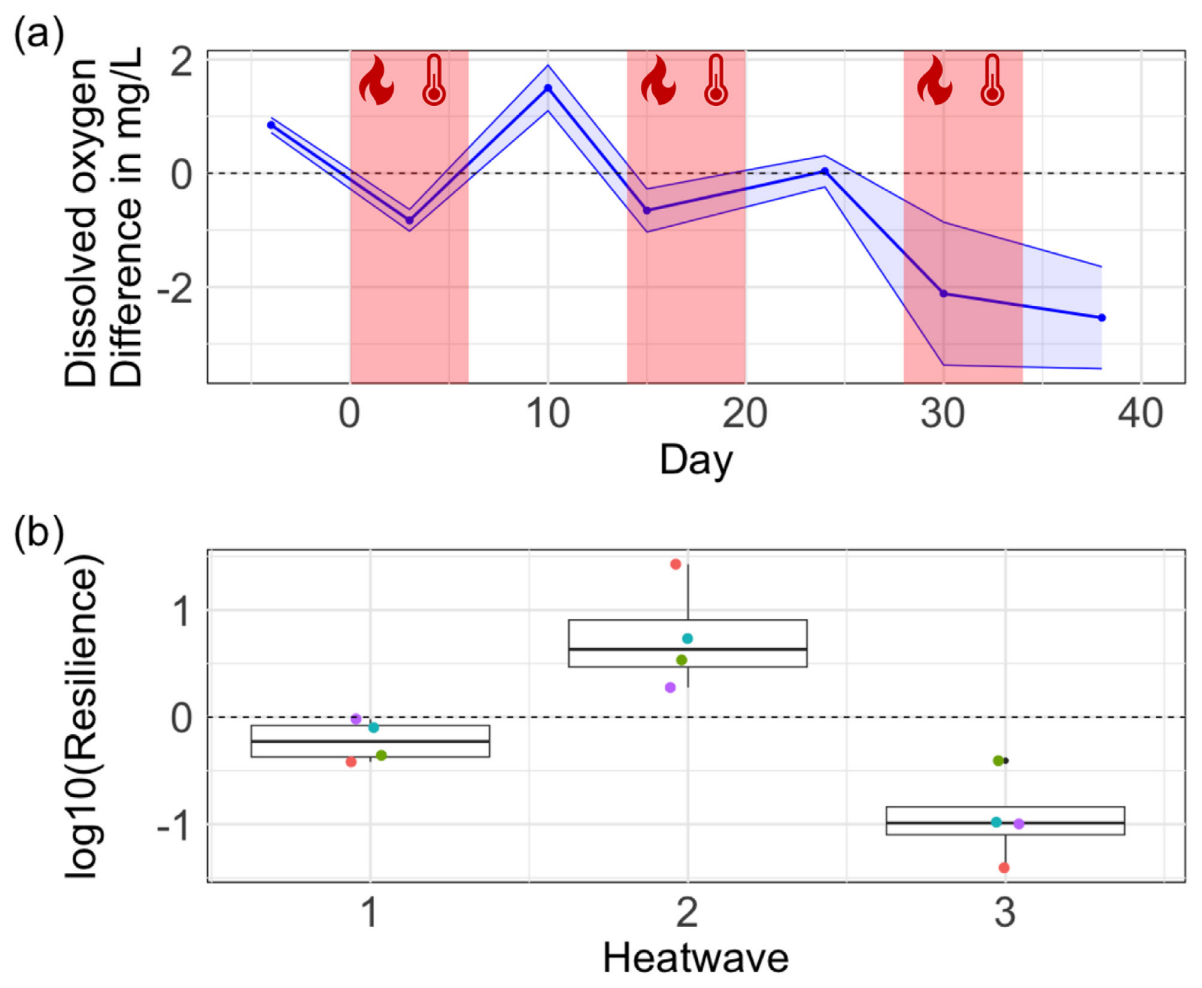
Dissolved oxygen dynamics over time. (a) shows the difference in DO between mesocosm undergoing the HW treatment and the control mesocosms (dashed line at zero). The red areas show the timing of the three heatwaves. (b) Boxplot of resilience of DO after each HW. Coloured dots represent the different mesocosms. Values of resilience > 0 mean increased resilience (HW and control mesocosms became more similar after a HW), whereas values of resilience < 0 mean decreased resilience (HW and control mesocosms became more dissimilar after a HW).

DO declined sharply during the first HW but recovered quickly to values higher than the control between the first and the second HW (Figure 2a). The second HW determined a similar decrease in DO, but with a less steep recovery trajectory. However, the third HW drastically decreased DO during the HW event. Following the last HW (day 38), DO further declined, showing no signs of recovery (Figure 2a). The trend was also confirmed by the calculated resilience, which became negative after the third HW (Figure 2b).

The LMM showed significant effects of the HW treatment (estimate: 0.12, 95% confidence interval of 0.013 to 0.23, *p*‐value: 0.046), and a significant interaction between HW and time (estimate: −0.008, 95% confidence interval of −0.013 to −0.003; *p*‐value: 0.003) suggesting a time‐dependent effect of the HWs on the DO concentration, that became more negative with time (Table A1). The post hoc analysis showed that there was a significant difference (*p* < 0.05) in the DO concentration between the control mesocosms and the mesocosms undergoing HWs on day −4, and 38 (Table A2).

### 3.3 Chlorophyll *a*

**FIGURE 3:**
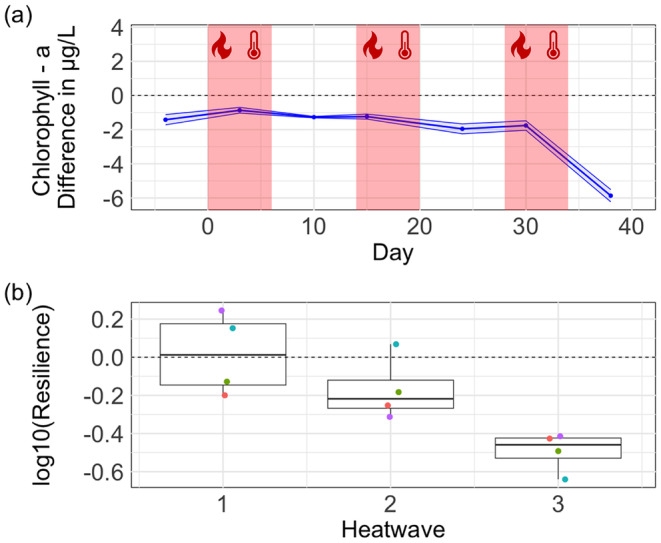
Chlorophyll *a* concentration dynamic over time. (a) shows the difference in Chlorophyll *a* between mesocosms undergoing the HW treatment and the control mesocosms (dashed line at zero). The red areas show the three heatwaves. (b) Boxplot of resilience of Chlorophyll *a* after each HW. Coloured dots represent the different mesocosms. Values of resilience > 0 mean increased resilience (HW and control mesocosms became more similar after a HW), whereas values of resilience < 0 mean decreased resilience (HW and control mesocosms became more dissimilar after a HW).

Chl *a* showed a slight decline after the first two HWs. However, after the third HW, the chl *a* concentration sharply declined in the HW mesocosms compared to control (Figure 3a). The resilience analysis also highlighted this trend. After the first and second HWs, resilience gradually declined. Yet, after the third HW, resilience showed the largest decline, with all HW mesocosms having large negative resilience values (Figure 3b).

LMM analysis showed that the HW treatment had a significant effect on the chlorophyll‐a concentration (estimate: −0.65, 95% confidence interval of −1.22 to −0.078; *p*‐value: 0.041; Table A3).

### 3.4 Phytoplankton Community Biomass and Composition

**FIGURE 4:**
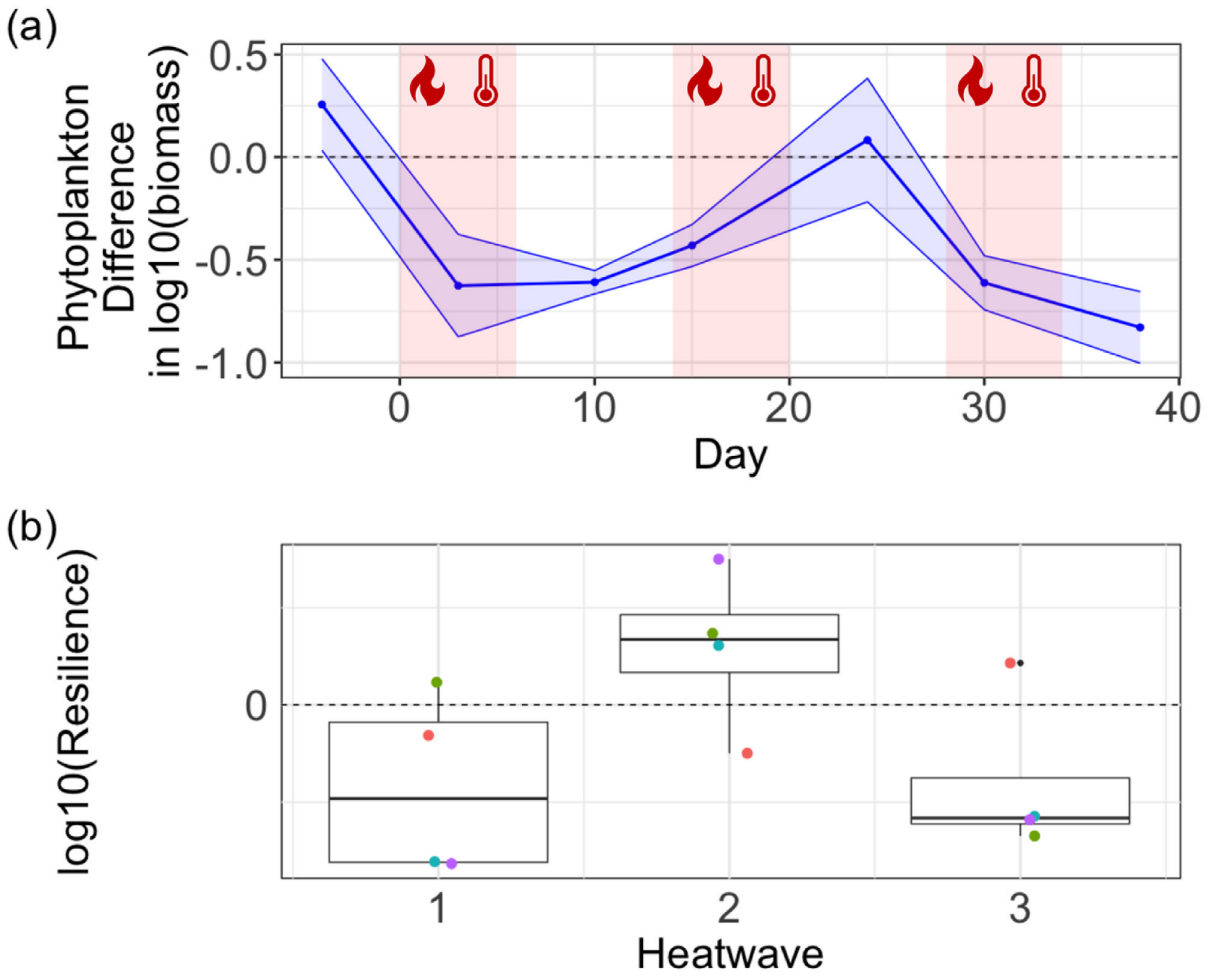
Phytoplankton biomass dynamic over time. (a) shows the difference in phytoplankton biomass between mesocosms undergoing the HW treatment and the control mesocosms (dashed line at zero). The red areas show the three heatwaves. (b) boxplot of resilience of phytoplankton biomass after each HW. Coloured dots represent the different mesocosms. Values of resilience > 0 mean increased resilience (HW and control mesocosms became more similar after a HW), whereas values of resilience < 0 mean decreased resilience (HW and control mesocosms became more dissimilar after a HW).

Phytoplankton biomass declined during and after the first HW, but recovered during and after the second HW. Eventually, another decline was noted during and after the third HW (Figure 4a). Yet, neither HW (estimate: −0.42, 95% confidence interval of −1.21 to 0.29; *p*‐value: 0.26; Table A4) nor time (estimate: 0.004, 95% confidence interval of −0.019 to 0.027; *p*‐value: 0.75; Table A4) had a significant effect on phytoplankton biomass. However, there was a marginally significant interaction between HW and time (*p*‐value = 0.085), indicating a time‐dependent effect of HWs on phytoplankton biomass (Table A4). The post hoc analysis showed that there was a significant difference (*p* < 0.05) in the phytoplankton biomass between the control mesocosms and the mesocosms undergoing HWs from day 10 onwards, with the mesocosms experiencing HW having a significantly lower phytoplankton biomass (Table A5). The resilience of phytoplankton biomass was reduced after the first HW. However, it recovered after the second HW, but became negative again after the third HW (Figure 4b).

**FIGURE 5:**
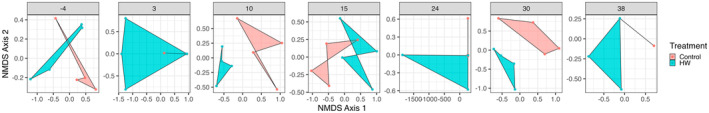
Non‐metric multidimensional scaling (NMDS) plots of phytoplankton community composition over time. Facets show different experimental days. Treatments are defined by colour. The dots present the replicated mesocosms.

The relative biomass of the different phytoplankton groups in the HW‐treated mesocosms showed a gradual turnover over the course of the experiment and gradually became more and more dissimilar to the control mesocosms (Figure 5). The increased compositional dissimilarity was confirmed by the PERMANOVA analysis, which highlighted a significant difference in community composition between the control and the HW‐exposed mesocosms on days 10 (*F* = 4.21, *p* = 0.027), 30 (*F* = 3.10, *p* = 0.032), and 38 (*F* = 2.55, *p* = 0.026; Table 1).

**TABLE 1:** Results of the PERMANOVA analysing the effects of the HWs on phytoplankton community composition in different days of the experiment.

Day	Residual	*F*	*R* ^2^	*p*
−4	6	0.895	0.130	0.367
3	6	3.091	0.340	0.067
**10**	**6**	**4.376**	**0.422**	**0.027**
15	6	1.766	0.227	0.15
24	6	0.783	0.115	0.569
**30**	**6**	**3.109**	**0.341**	**0.032**
**38**	**6**	**2.553**	**0.298**	**0.026**

*Note:* Significant differences in community compositon (*p* < 0.05) are reported in bold.

**FIGURE 6:**
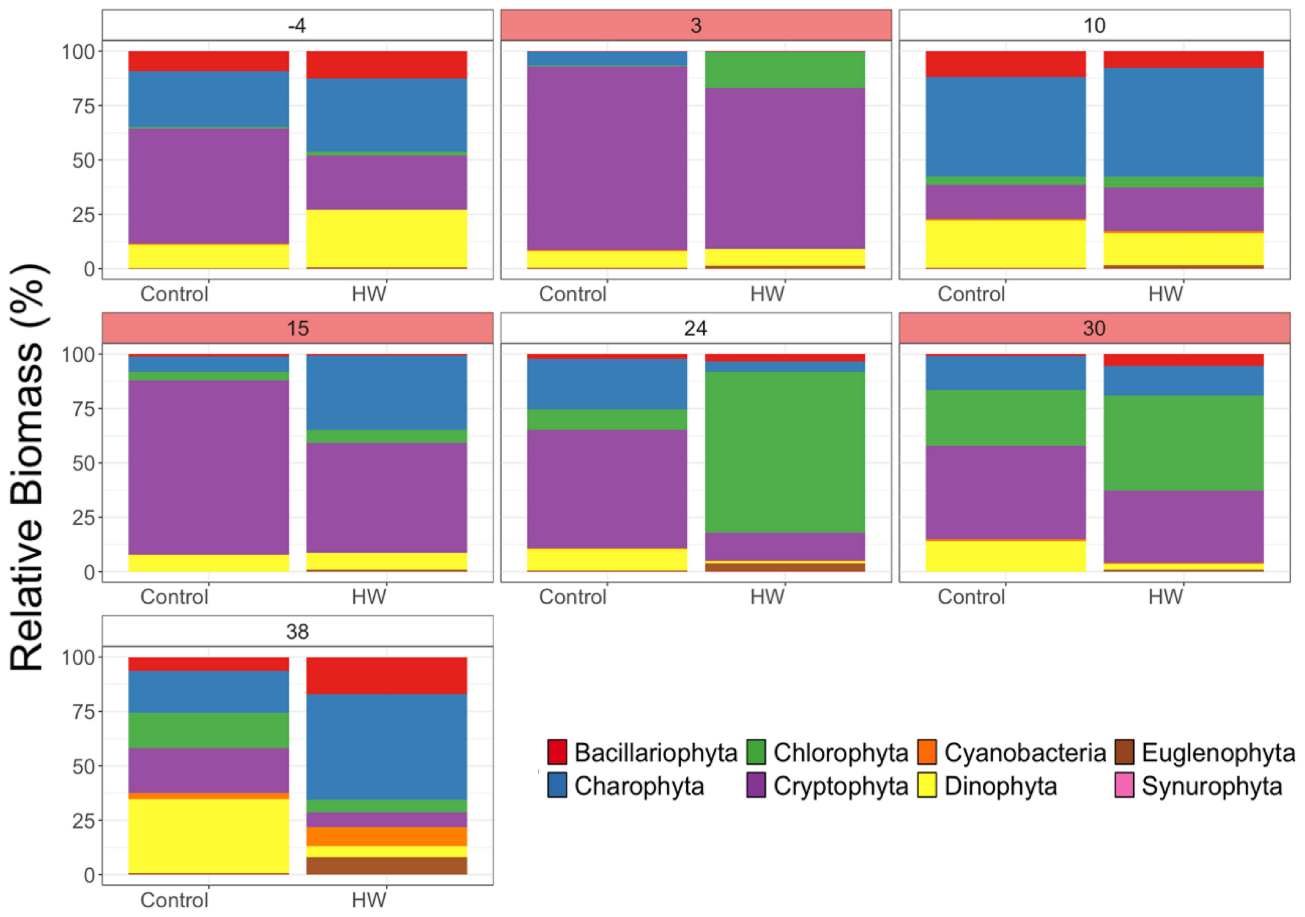
Mean relative biomass of different phytoplankton taxa in the control and the mesocosms exposed to HWs in different time points of the experiment. Day facets in white represent non‐HW days, whereas light red facets represent HW days.

Significant differences on day 10 were related to a significant decline in Charophyta, Cryptophyta, and Bacillariophyta biomass in the HW treatment compared to the control (Figure 6; SIMPER; Table A6). On day 30, the compositional change was driven by a significant decline in Cryptophyta (Figure 6; SIMPER; Table A6), while at day 38 there was a significant reduction in Cryptophyta and Chlorophyta biomass in the mesocosms exposed to the HWs (Figure 6; SIMPER; Table A6).

### 3.5 Zooplankton Community Biomass and Composition

**FIGURE 7:**
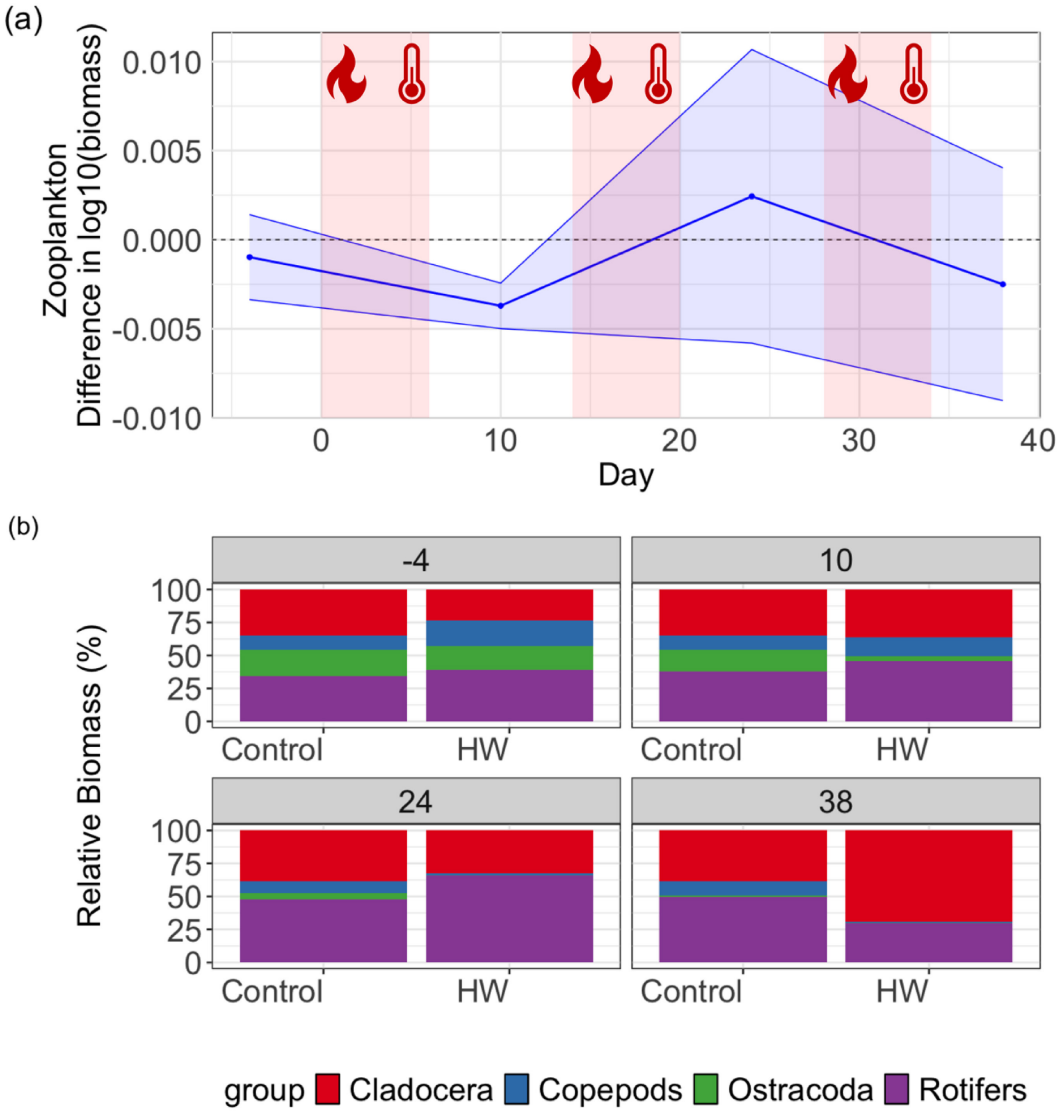
Zooplankton biomass dynamic over time. (a) Shows the difference in zooplankton biomass between mesocosms undergoing the HW treatment and the control mesocosms (dashed line at zero). The red areas show the three heatwaves. (b) Mean relative biomass of different zooplankton taxa in the control and the mesocosms exposed to HWs in different sampling days of the experiment.

The zooplankton biomass was less affected by the HWs than phytoplankton biomass, showing only an initial decline after the first HW (Figure 7). Later, the variability between replicates was large, but the general trend showed an increase after the second HW, and a subsequent decline after the third HW. The LMM analysis shows a significant effect of time on zooplankton biomass (estimate: 0.006, 95% confidence interval from 0.004 to 0.009, *p*‐value < 0.001; Table A7).

Community composition was also not significantly affected by the HWs, as showed by the PERMANOVA analysis (Table A8). However, a general increase in Cladocera and a decrease of Copepoda could be noted after the third HW (Figure 7).

## 4 Discussion

Our experiment shows profound effects of recurring HWs on the resilience of aquatic communities. Our findings align with the growing body of literature reporting that HWs have significant detrimental effects on aquatic ecosystems (Hermann, Peeters, and Van den Brink 2023; Hermann et al. 2024; Polazzo et al. 2022; Ross et al. 2022). Critically, we show that a series of three, repeated HWs can erode the resilience of phytoplankton communities in freshwater ecosystems. The observed trend in dissolved oxygen (DO) levels suggests a pattern of critical slowing down, indicated by a gradual reduction in recovery after repeated disturbances, which eventually determined an erosion of resilience (Veraart et al. 2012). It is important to note that the positive resilience value following the second heatwave was solely driven by the high dissolved oxygen (DO) concentration on day 10, which was much higher than DO in control mesocosms. This led to a positive resilience measurement after the second HW, despite the DO concentration on day 24 being similar to that in the control mesocosms. Overall, we observed a gradual decline in DO levels after each heatwave (Figure 2a).

HWs can cause abrupt increases in DO consumption (Yvon‐Durocher et al. 2012), ultimately modifying the hourly amplitude of the balance between carbon dioxide and DO without altering the diurnal frequency of the lake's metabolic cycle. The increased DO consumption caused by the HWs, together with the heat‐driven reduced photosynthetic activity, determined an overall decline in DO after each HW and reduced its resilience.

Chlorophyll *a*, on the other hand, showed a decline in both concentration and resilience after each HW. The steady decline in resilience after each HW event suggests an impaired recovery potential for chlorophyll *a*, consistent with the critical slowing down hypothesis. A recent experiment found that chlorophyll *a* increased after exposure to a first HW, but then returned to control levels after a second HW (Huỳnh et al. 2024). The different responses of chlorophyll a in Huỳnh et al. (2024) and our study may be related to the intensity of the HW treatment. Indeed, we applied a temperature difference of +8°C in the HW treatment, whereas Huỳnh et al. (2024) applied a difference of +6°C. Additionally, the maximum temperature in the HW mesocosms in our study was 36°C, whereas 32°C was not reached in Huỳnh et al. (2024). The difference in absolute temperature may have determined the larger decline and loss of resilience in chlorophyll a in our study, as the increased temperature stress could have led to a larger reduction in the photosynthetic activity of phytoplankton. Yet, our results align with those of Veraart et al. (2012), who found a gradual decline in photosynthetic activity in a phytoplankton species exposed to an increasing level of stress.

Phytoplankton biomass exhibited a less clear response. During and after the first HW, phytoplankton biomass declined. The biomass decline was associated with a significant compositional change after the first HW (day 10), and a loss of resilience. Yet, after the second HW, phytoplankton biomass recovered to control levels, and no compositional difference was noted between the control and the HW mesocosms on day 24. This similarity suggests that after the first HW, which caused a significant change in composition, the phytoplankton community composition recovered and was indistinguishable from the control. This recovery in biomass and composition determined an increase in resilience, suggesting a possible community rescue. However, the third HW determined a decline in biomass which was associated with a significant compositional turnover and with reduced resilience. Particularly, towards the end of the experiment, the compositional dissimilarity was driven by a significant reduction in the biomass of Cryptophyta, Dinophyta, and Chlorophyta in the HW treatment. The new community composition did not promote stress‐tolerant species capable of maintaining phytoplankton biomass and increasing resilience, as evidenced by the decline of both biomass and resilience. Although community rescue is usually linked to a strong compositional change, this compositional change should determine an increased resistance to stress and a consequent ability to maintain community biomass and restore resilience (Fugère et al. 2020). Since we found the opposite (i.e., compositional change determined a decline in resistance to following HW and a biomass decline), we excluded the idea that a rescue process occurred in our experiment.

On the contrary, the increased compositional dissimilarity, linked to the reduced resilience, supports the critical slowing down hypothesis. Critical transitions to alternative stable states are often related to dramatic shifts in composition (Bertani, Primicerio, and Rossetti 2016; Meunier, Hacker, and Menge 2024; Wernberg et al. 2016). The classic example is the shift from the clear state of shallow lakes dominated by macrophyte to a turbid water state dominated by phytoplankton (Scheffer 2009). The compositional shift is a common feature of critical transitions across ecosystems and has been reported in marine (Meunier, Hacker, and Menge 2024) as well as terrestrial systems (Eby et al. 2017). Hence, our study aligns with the body of literature describing strong compositional shifts, which relate to dramatic changes in community biomass, as the main driver of critical transitions (Eby et al. 2017; Meunier, Hacker, and Menge 2024).

Ultimately, the response of phytoplankton to a HW depends on the thermal sensitivity of the species forming the community (Polazzo et al. 2022) and on the ecological interactions (re‐)established during and after the HW (Huỳnh et al. 2024; Polazzo et al. 2023; Seifert, Weithoff, and Vos 2015). In our experiment, although the temperature difference between the HW treatment and the control was +8°C for all HW events, the temperature in the control increased as we progressed from April to July. This resulted in the HWs having an increasing absolute temperature. The strong decline in biomass and chlorophyll *a* after the third heatwave may have been determined by the higher proportion of species unable to cope with the exaggerated thermal stress of another, stronger HW. Despite the cumulative stress caused by recurrent HWs, the intensity of an HW has been shown to affect planktonic communities differently, not only during, but especially after, the HW event (Seifert, Weithoff, and Vos 2015).

In our experiment, the change in the zooplankton activity arises as another factor that may have contributed to the decline in phytoplankton biomass and chlorophyll‐a. Although, the recurring HWs did not significantly affect zooplankton biomass or composition, at the end of the experiment, the mesocosms exposed to the recurring HWs, had a larger proportion of Cladocera compared to the control mesocosms. The grazing efficiency of Cladocera on suspended algae is significantly larger than that of copepods or rotifers (Sommer et al. 2002). The increase in Cladocera may have resulted in increased grazing, and thus stronger top‐down control on primary producers, contributing to the overall decline in phytoplankton biomass. Huỳnh et al. (2024) found a higher relative abundance of copepods in mesocosms exposed to two consecutive HWs, leading to an overall weaker top‐down control (Huỳnh et al. 2024). However, an increase in small cladocerans was reported in another experiment where a zooplankton community was exposed to a HW of similar duration and intensity (Roth et al. 2022).

Overall, we highlight that it is not necessary for a community to tip in order to show a slowing down in recovery. van Nes and Scheffer (2007) suggested that critical slowing down may not only be related to a critical transition or tipping points but may also generically indicate a reduced tolerance of the system to repeated perturbations. Critical slowing down may thus provide important information in cases where the threshold leading to a critical transition has not yet been reached, thus working as an EWS, and may be informative for systems that do not have multiple stable states at all.

In conclusion, our study shows that the repeated stress caused by increasingly stronger HWs led to a drastic change in the composition of the phytoplankton community. The new compositional configuration was unable to maintain and/or recover photosynthetic activity or biomass levels as in undisturbed systems. This suggests an overall decrease in the resilience of aquatic communities and ecosystems to subsequent perturbations, supporting the critical slow‐down hypothesis. These results force us to evaluate the consequences of climate change‐induced extreme weather events on community and ecosystem functioning, particularly as such extreme events become more recurrent and severe in the future.

## Author Contributions

**Francesco Polazzo:** conceptualization (equal), formal analysis (equal), investigation (equal), methodology (equal), writing – original draft (equal). **Markus Hermann:** investigation (equal), methodology (equal), writing – review and editing (equal). **Melina Crettaz‐Minaglia:** investigation (equal), methodology (equal), writing – review and editing (equal). **Andreu Rico:** conceptualization (equal), funding acquisition (equal), investigation (equal), methodology (equal), project administration (equal), supervision (equal), writing – review and editing (equal).

## Conflicts of Interest

The authors declare no conflicts of interest.

## Data Availability

Data and code to reproduce the analysis and figures is available at https://github.com/FrancescoPola/rescue_critical_slowing, and have been permanently deposited in Zenodo (Polazzo 2024).

## Appendix

**TABLE A1:** Results of the LMM analysing the effects of HW and time on the DO concentration.

Effect	Term	Estimate	2.5%	97.5%	*p*
Fixed	(Intercept)	2.418	2.342	2.494	0.000
**Fixed**	**TreatmentHW**	**0.121**	**0.013**	**0.228**	**0.046**
Fixed	Day	0.001	−0.002	0.005	0.483
**Fixed**	**TreatmentHW:Day**	**−0.008**	**−0.013**	**−0.003**	**0.003**

*Note:* Significant results (*p* < 0.05) are reported in bold.

**TABLE A2:** Results of the post hoc test (estimated marginal means (EMMs) analysis) of the LMM assessing the effects of HWs and time on the DO concentration.

Contrast	Day	Estimate	SE	df	*t*‐ratio	*p*	
**Control—HW**	**−4**	**−0.153**	**0.064**	**24.889**	**−2.376**	**0.026**	
Control—HW	10	−0.040	0.042	8.521	−0.950	0.368	
Control—HW	24	0.073	0.042	8.521	1.721	0.121	
**Control—HW**	**38**	**0.185**	**0.064**	**24.889**	**2.882**	**0.008**	

*Note:* Significant results (*p* < 0.05) are reported in bold.

**TABLE A3:** Results of the LMM analysing the effects of HW and time on chlorophyll *a*.

Effect	Term	Estimate	2.5%	97.5%	*p*
Fixed	(Intercept)	0.657	0.252	1.062	0.005
**Fixed**	**TreatmentHW**	**−0.650**	**−1.222**	**−0.078**	**0.041**
Fixed	Day	0.011	−0.008	0.029	0.274
Fixed	TreatmentHW:Day	−0.020	−0.046	0.007	0.157

*Note:* Significant results (*p* < 0.05) are reported in bold.

**TABLE A4:** Results of the LMM analysing the effects of HW and time on phytoplankton biomass.

Effect	Term	Estimate	2.5%	97.5%	*p*
Fixed	(Intercept)	2.445	2.229	2.662	0.000
Fixed	TreatmentHW	−0.180	−0.487	0.126	0.263
Fixed	Day	0.002	−0.008	0.012	0.752
Fixed	TreatmentHW:Day	−0.013	−0.027	0.001	0.085

**TABLE A5:** Results of the post hoc test (estimated marginal means (EMMs) analysis) of the LMM assessing the effects of HWs and time on phytoplankton biomass.

Contrast	Day	Estimate	SE	df	*t*‐ratio	*p*
Control—HW	−4	0.129	0.183	37.643	0.702	0.487
Control—HW	3	0.219	0.143	20.521	1.531	0.141
**Control—HW**	**10**	**0.310**	**0.113**	**8.935**	**2.736**	**0.023**
**Control—HW**	**15**	**0.375**	**0.103**	**6.155**	**3.636**	**0.010**
**Control—HW**	**24**	**0.491**	**0.116**	**9.827**	**4.230**	**0.002**
**Control—HW**	**30**	**0.569**	**0.143**	**20.192**	**3.993**	**0.001**
**Control—HW**	**38**	**0.673**	**0.188**	**39.467**	**3.571**	**0.001**

*Note:* Significant results (*p* < 0.05) are reported in bold.

**TABLE A6:** Results of the SIMPER analysing the effects of the HWs on phytoplankton taxa biomass over the course of the experiment.

Taxonomic groups	Average	sd	Ratio	ava	avb	cumsum	*p*
Day −4							
Charophyta	0.206	0.171	1.207	293.749	99.457	0.371	0.401
Dinophyta	0.133	0.131	1.013	228.716	42.859	0.611	0.083
Cryptophyta	0.133	0.104	1.273	215.925	206.102	0.850	0.319
Bacillariophyta	0.072	0.036	2.011	109.057	35.663	0.980	0.177
Chlorophyta	0.007	0.006	1.157	14.110	2.809	0.992	0.728
Euglenophyta	0.003	0.004	0.695	6.179	0.373	0.997	0.428
Cyanobacteria	0.002	0.002	0.768	0.559	1.160	1.000	0.875
Synurophyta	0.000	0.000		0.000	0.000	1.000	0.001
Day 3							
Cryptophyta	0.513	0.289	1.779	75.579	230.067	0.741	0.051
Dinophyta	0.076	0.091	0.834	8.011	21.363	0.850	0.332
Chlorophyta	0.047	0.081	0.584	16.870	1.239	0.919	0.908
**Charophyta**	**0.042**	**0.062**	**0.682**	**0.342**	**17.338**	**0.980**	**0.037**
**Cyanobacteria**	**0.007**	**0.010**	**0.642**	**0.000**	**1.684**	**0.990**	**0.001**
Euglenophyta	0.005	0.006	0.766	1.439	0.804	0.997	0.944
Bacillariophyta	0.002	0.003	0.705	0.186	0.747	1.000	0.370
Synurophyta	0.000	0.000		0.000	0.000	1.000	0.001
Day 10							
**Charophyta**	**0.272**	**0.237**	**1.147**	**32.657**	**135.963**	**0.407**	**0.031**
Dinophyta	0.148	0.121	1.224	9.747	65.023	0.628	0.053
**Cryptophyta**	**0.146**	**0.108**	**1.347**	**13.064**	**45.836**	**0.846**	**0.001**
**Bacillariophyta**	**0.072**	**0.078**	**0.926**	**5.035**	**34.741**	**0.954**	**0.001**
Chlorophyta	0.023	0.025	0.911	3.339	11.008	0.988	0.184
Cyanobacteria	0.005	0.007	0.803	0.550	2.366	0.996	0.946
Euglenophyta	0.003	0.004	0.713	1.014	0.765	1.000	0.975
Synurophyta	0.000	0.000		0.000	0.000	1.000	0.001
Day 15							
Cryptophyta	0.430	0.249	1.729	68.053	307.782	0.687	0.120
Charophyta	0.091	0.089	1.021	46.209	26.590	0.832	0.949
Dinophyta	0.063	0.077	0.808	10.681	29.374	0.932	0.062
Chlorophyta	0.030	0.024	1.232	8.345	15.295	0.980	0.403
Bacillariophyta	0.009	0.008	1.069	0.773	4.510	0.993	0.113
Euglenophyta	0.003	0.004	0.626	1.057	0.000	0.998	0.893
**Cyanobacteria**	**0.001**	**0.001**	**2.037**	**0.002**	**0.639**	**1.000**	**0.001**
Synurophyta	0.000	0.000		0.000	0.000	1.000	0.001
Day 24							
Chlorophyta	0.232	0.325	0.713	456.690	21.977	0.393	0.866
Cryptophyta	0.152	0.151	1.008	80.832	125.624	0.652	0.740
Charophyta	0.106	0.161	0.654	29.724	53.875	0.831	0.543
Dinophyta	0.049	0.050	0.990	6.142	23.099	0.915	0.078
Euglenophyta	0.029	0.037	0.785	23.128	1.110	0.964	0.365
Bacillariophyta	0.019	0.011	1.748	21.166	4.900	0.996	0.459
Cyanobacteria	0.003	0.003	0.966	1.106	0.848	1.000	0.619
Synurophyta	0.000	0.000		0.000	0.000	1.000	0.001
Day 30							
**Cryptophyta**	**0.332**	**0.264**	**1.256**	**31.725**	**165.021**	**0.452**	**0.001**
Chlorophyta	0.180	0.169	1.066	41.734	99.096	0.697	0.933
Charophyta	0.110	0.158	0.696	12.816	59.473	0.847	0.306
Dinophyta	0.095	0.090	1.055	2.670	53.407	0.976	0.058
Bacillariophyta	0.010	0.009	1.016	5.289	3.337	0.989	0.936
Cyanobacteria	0.006	0.005	1.127	0.250	3.758	0.998	0.113
Euglenophyta	0.002	0.003	0.519	0.835	0.000	1.000	0.971
Synurophyta	0.000	0.000		0.000	0.000	1.000	0.001
Day 38							
**Cryptophyta**	**0.230**	**0.162**	**1.420**	**4.567**	**100.140**	**0.304**	**0.001**
Dinophyta	0.173	0.194	0.895	3.471	167.298	0.533	0.147
**Chlorophyta**	**0.130**	**0.166**	**0.783**	**3.894**	**80.553**	**0.704**	**0.039**
Charophyta	0.127	0.055	2.308	32.507	93.918	0.872	0.967
Bacillariophyta	0.059	0.047	1.260	11.421	31.128	0.950	0.429
Cyanobacteria	0.027	0.024	1.147	5.863	14.455	0.986	0.967
Euglenophyta	0.011	0.010	1.004	5.281	3.097	1.000	0.929
Synurophyta	0.000	0.000		0.000	0.000	1.000	0.001

*Note:* The column “Average” represents the average contribution of each taxon to the overall dissimilarity between groups. “sd” is the standard deviation of the contributions of each species to the dissimilarity. “ratio” is the ratio of the average contribution to the standard deviation (average/sd). This indicates the consistency of the species' contribution to the dissimilarity. Higher ratios suggest more consistent contributions. “ava” is the average biomass of each species in group A (i.e., HW). “avb” is the average biomass of each species in group B (i.e., Control). “cumsum” is the cumulative sum of the contributions of species to the overall dissimilarity, expressed as a fraction. This shows the cumulative proportion of the total dissimilarity accounted for by the species up to that row in the table. *p*‐value are resulting from permutation test. Significant results (*p* < 0.05) are reported in bold.

**TABLE A7:** Results of the LMM analysing the effects of HW and time on zooplankton biomass.

Effect	Term	Estimate	2.5%	97.5%	*p*
Fixed	(Intercept)	0.128	0.063	0.192	0.001
Fixed	TreatmentHW	0.014	−0.050	0.079	0.678
**Fixed**	**Day**	**0.006**	**0.004**	**0.009**	**> 0.001**
Fixed	TreatmentHW:Day	0.000	−0.003	0.002	0.816

*Note:* Significant results (*p* < 0.05) are reported in bold.

**TABLE A8:** Results of the PERMANOVA analysing the effects of the HWs on zooplankton community composition in different days of the experiment.

Day	df_model	df_residual	*F*	*R* ^2^	*p*
−4	1	6	0.241	0.039	0.670
10	1	6	0.547	0.084	0.732
24	1	6	1.228	0.170	0.194
38	1	6	0.713	0.106	0.609
